# Service and Treatment Factors as Predictors of Satisfaction with Mental Health Services Among Service Users with Psychosis

**DOI:** 10.1007/s10597-024-01418-9

**Published:** 2024-12-10

**Authors:** Regina Skar-Fröding, Hanne Kristin Clausen, Eva Biringer, Torleif Ruud, Jurate Šaltyte Benth, Mina Veland, Kristin S. Heiervang

**Affiliations:** 1 R&D Department, Division of Mental Health Services, Akershus University Hospital, P.O. Box 1000, Lørenskog, 1478 Norway; 2 Norwegian National Advisory Unit on Concurrent Substance Abuse and Mental Health Disorders and Mental Health Division, Innlandet Hospital Trust, Brumunddal, Norway; 3 Department of Research and Innovation, Helse Fonna HF, Stord Hospital, Stord, Norway; 4 Institute of Clinical Medicine, University of Oslo, Oslo, Norway; 5 Institute of Clinical Medicine, University of Oslo, Campus Ahus, P.O. Box 1171, Blindern, 0318 Norway; 6 Health Services Research Unit, Akershus University Hospital, P.O. Box 1000, Lørenskog, 1478 Norway

**Keywords:** Satisfaction, Mental health services, Psychosis

## Abstract

Satisfaction with services among service users is an important aspect of quality in mental health care. This prospective study investigated associations between service and treatment factors at baseline and satisfaction with services at 18-month follow-up among service users with psychosis in specialist mental health services. Data were collected from 119 service users with psychosis and their clinicians from 26 clinical sites across Norway at baseline and after 18 months. Satisfaction with services was measured using the Client Satisfaction Questionnaire-8 (CSQ-8). Linear mixed models with random intercepts for units were estimated to test the association between service- and treatment-related predictors and the CSQ-8. Participating in the Individual Placement and Support program or other interventions to promote work or study, receiving well-coordinated services, and receiving helpful assistance from a general practitioner at baseline were positively associated with satisfaction with services at 18-month follow-up. The present results suggest that receiving integrated and well-coordinated services and targeting the goal of facilitating employment and study opportunities is important for satisfaction with services among service users with psychosis.

## Introduction

Service users’ satisfaction with services has become an important quality indicator and outcome in mental health care (Priebe & Miglietta, 2019). Service user satisfaction has even been shown to be a more reliable indicator of service quality than quality ratings from a referrer or clinician (Shipley et al., 2000). Historically, reports from service users with psychosis have been disregarded because of the belief that they lack the insight and the ability needed to evaluate mental health services and provide valid feedback (Ruggeri et al., 2003). Nowadays, however, satisfaction with mental health care services among service users with psychosis is an important topic increasingly being investigated by researchers (Reininghaus & Priebe, 2012). Satisfaction with services is also an important part of patient-centered care, which is an approach to mental health services that promotes patient preferences and values (Carey Prof, 2016). Patient- and service user-centered care is emphasized by both international (World Health Organization, 2013) and national (Sykehustalen, 2017) political guidelines, as well as by user organizations (Erfaringskompetanse.no, 2015).

Among service users with psychosis, higher satisfaction with mental health services is associated with clinical outcomes such as a reduction in positive symptoms at follow-up (Vermeulen, Schirmbeck, van Tricht, de Haan, & investigators, 2018). In addition, a lower level of satisfaction with services has been shown to be associated with greater illness severity, more involuntary admissions and unmet needs (Ruggeri et al., 2003). A cross-sectional study investigating predictors of satisfaction with care among service users with schizophrenia in community mental health teams (CMTHs) reported that higher service user satisfaction was associated with a lower level of disability, a longer duration of treatment in CMTHs, younger age and lower education (Hat et al., 2022).

Satisfaction with services can be influenced by both individual factors, such as personal characteristics and clinical symptoms, and treatment/service factors (Woodward et al., 2017). For example, a randomized controlled trial found that integrated care was associated with higher satisfaction with treatment compared with standard care among service users who had experienced their first episode of psychosis (Petersen et al., 2005). In a study by Hat et al. (2022), satisfaction with care was lowest when family involvement was insufficient in the therapeutic process, and if/when the availability of the therapeutic team was insufficient. Lower satisfaction with care was also associated with a negative experience of psychiatric hospitalization (especially involuntary hospitalization) and lack of improvement in work ability. In addition, a literature review investigating satisfaction with pharmacological treatment reported that service users’ dissatisfaction with antipsychotic medication was influenced by multiple factors, including drug side effects, a lack of involvement in treatment planning or decision-making and lack of involvement of family members in care planning (Chue, 2006). A naturalistic trial study found that higher number of prescribed drugs were linked to lower satisfaction among service users with schizophrenia (Köhler et al., 2015).

In recent years, more emphasis has been placed on the importance of providing comprehensive and well-coordinated mental health services (World Health Organization, 2013). This has also been emphasized in the Coordination Reform in Norway, which aims to improve collaboration between service levels (Ministry of Health and Care Services, 2009). The capacity of different professionals and services to provide comprehensive and well-coordinated services is a key challenge in achieving continuity of care (Haggerty et al., 2003; Nicaise et al., 2020). However, the relationship between comprehensive and continuous care and mental health patient outcomes, including satisfaction with services, remains inconsistent (Puntis et al., 2015). Identifying health service factors that impact perceived satisfaction has important clinical implications for mental health services for patients with psychosis, as satisfaction with services is an important aspect of both quality of care (Reininghaus & Priebe, 2012) and patient-centered care (Carey Prof, 2016).

At the individual level, previous studies have found the strongest associations between characteristics such as symptoms or other service user characteristics and satisfaction with mental health services (Bener & Ghuloum, 2013; Bird et al., 2020; Bjørngaard et al., 2007; Boydell et al., 2012; Vermeulen et al., 2018). However, most studies investigating the associations between individual- or service-level characteristics, and service users’ satisfaction with services have been cross-sectional in design. Studying the prospective associations between potential predictors, and service users’ satisfaction with services allows for higher degree of inference regarding the directions of the associations. Therefore, we conducted the present longitudinal study on a sample of patients with psychosis who were using specialist mental health care services.

### Aims

This study aimed to investigate the prospective associations between service and treatment factors at baseline and satisfaction with services at 18-month follow-up. We addressed the following research questions: ‘What types of treatments received at baseline are associated with service users’ satisfaction with services at 18-month follow-up?’, ‘Is comprehensive treatment associated with satisfaction with services at 18-month follow-up?’ and ‘What aspects of community services and collaboration with primary care are associated with satisfaction with services?’.

## Method

### Context

In Norway, public mental health care services are structured into two main levels: municipal health and care services (primary care) and specialist health services (secondary and tertiary care), both of which are responsible for providing mental care for citizens. Specialist health services offer comprehensive specialized care, including inpatient treatment, outpatient treatment and ongoing care. Individuals with severe mental illness often receive specialist health services. General practitioners (GPs) typically refer service users to a specialist mental health service. Most often patients are referred to a community mental health center (CMHC; i.e. secondary care), for outpatient or inpatient treatment. Patients can also be referred to a psychiatric hospital directly (tertiary care).

### Design

This prospective study analyzed data from baseline and 18-month follow-up from the Norwegian research project ‘A Pairwise Randomized Study on Implementation of Guidelines and Evidence-based Treatments of Psychoses’ (ClinicalTrials NCT03271242). This study was approved by the Regional Committee for Medical and Health Research Ethics (REK Sørøst B 2015/2169) and followed the principles of the Declaration of Helsinki.

### Setting and Sample

A total of 325 service users and their clinicians from 32 clinical units completed questionnaires at baseline. Due to discharges from mental health services and dropouts from the study, the final sample was reduced to 119 service users and 113 clinicians, representing service user-clinician dyads, from 26 clinical units at 18-month follow-up.

The participants were service users with psychosis receiving mental health services recruited from six regional health authorities in Norway, including three university hospitals. The clinical units and hospital departments comprised outpatient clinics, day units, mobile teams, and inpatient wards. The following inclusion criteria were used: age ≥ 16 years and diagnosed with and/or treated for psychosis at a service unit specializing in psychosis. The only exclusion criterion was the inability to understand and respond to the questionnaires in Norwegian.

### Procedures

Clinicians from the participating mental health units recruited eligible service users who were either already in contact with the clinic during the study period or newly referred. The clinicians conducted clinical assessments, and questionnaires were administered to the service users by either the secretary or other staff members at the unit. Service users completed the questionnaires either at the clinic, where they were provided with a designated place to sit, or at home. After completing the questionnaires, they were sealed in an envelope and returned to the clinic. The recruitment phase was from June 2016 to March 2017, and only participants who provided written informed consent were included in the study.

To increase the relevance of the study from the service user perspective, an expert by experience has actively contributed to the planning, selection of relevant covariates, interpretation of results, and discussions presented in this paper.

### Measures

#### Outcome Measure

The Client Satisfaction Questionnaire-8 (CSQ-8) (Attkisson & Greenfield, 1996) is a self-report questionnaire that assesses patients’ overall satisfaction with the services they receive. The CSQ-8 gauges general satisfaction through eight scaled items, with responses ranging from 1 (poor) to 4 (excellent), resulting in a total score range of 8 to 32. The CSQ-8 is composed of the following eight items: ’How would you rate the quality of service received?’, ’Did you get the kind of service that you wanted?’, ’To what extent has our programme met your needs?’, ’If a friend were in need of similar help, would you recommend our programme to him or her?’, ’How satisfied are you with the amount of help you have received?’, ’Have the services you received helped you to deal more effectively with your problems?’, ’In an overall, general sense, how satisfied are you with the service you have received?’, and ’If you were to seek help again, would you come back to our programme?’

The CSQ-8 has demonstrated robust psychometric properties (Attkisson & Zwick, 1982) and has been used in a range of settings and populations (De Wilde & Hendriks, 2005; Pedersen et al., 2022). In the sample of the present study, intra-scale consistency was acceptable with a Cronbach’s alpha of 0.91.

#### Predictors

##### Treatment Received

Clinicians completed a checklist at baseline assessing whether the service users had received specific treatments or interventions during the past 6 months (Yes/No). The following interventions and treatments were assessed: Individual Placement and Support (IPS)/interventions to promote work or study, cognitive therapy, Illness Management and Recovery (IMR), organized physical activity, examination/follow-up for physical illness, family involvement and antipsychotic medication. The family involvement variable encompasses whether the participant had participated in either family meetings or group interventions for families. The antipsychotic medication variable was dichotomized as either having 0 or 1 type of antipsychotic medication versus having 2 or more, based on previous research showing that the number of prescribed drugs correlated negatively with satisfaction among service users with schizophrenia (Köhler et al., 2015).

##### Comprehensive Care

The comprehensive care variable consisted of the number of treatments received, ultimately resulting in a score between 0 and 7.

##### Community Treatment Order (CTO) Status

Information on whether the participants were subject to a CTO (Yes/No) at the time of inclusion in the study was included as a potential confounding factor.

##### Community Services and Collaboration Between Specialized Mental Health Care and Primary Care

Service users responded to the following statements on a scale ranging from 1 (totally disagree) to 5 (totally agree), with higher scores indicating more or better assistance: ’I have received helpful assistance from my general practitioner’ and ‘The services I have received are well-coordinated’.

Clinicians answered questions on whether a responsibility group was established around the service user (Yes/No) and whether the service user had a community coordinator (Yes/No). In several countries, coordinators or case-managers are roles that have been developed to try to ensure that people with severe mental illness receive consistent and coordinated care (Tansella et al., 2014).

The participants’ extent of contact with their GP was included as a variable. This information was based on register data from the Norway Control and Payment of Health Reimbursement register. This variable consists of the number of consultations between the GP and the patient, including home visits, for the period of 6 months before and after inclusion.

### Analysis

The characteristics of the participant were presented as means and standard deviations (SDs) for continuous variables, and frequencies and percentages for categorical variables. Due to the high dropout rate from baseline to follow-up, the drop-outs (*n* = 206) were compared with the 119 participants subjected to analysis. The differences between groups with regard to continuous variables (CSQ-8, comprehensive care, helpful assistance from GP, well-coordinated services, extent of contact with GP, the Symptoms and Functioning dimensions of the Global Assessment of Functioning Scale and age) were assessed by an independent sample t-test, while the differences between groups with regard to categorical variables (IPS/interventions to promote work or study, cognitive therapy, IMR, organized physical activity, examination/follow-up for physical illness, family involvement, antipsychotic medication, responsibility team, community coordinator, being under a CTO and gender) were compared using a χ2-test.

Because the study participants came from different clinical sites and health authorities, a hierarchical structure may have been present in the data. Cluster effects on both the site and health authority level were assessed using intra-class correlation coefficients. Three linear mixed models were estimated to assess the associations between service satisfaction (CSQ-8) at 18-month follow-up as the outcome and the preselected covariates at baseline. Model 1 consisted of treatments received for the last six months before/until baseline as covariates, Model 2 comprehensive care at baseline and Model 3 covariates on community services and collaboration with primary care. Adjusted and unadjusted models were estimated. All adjusted models were controlled for CTO status as a potential confounder. Random intercepts for sites were included in all models. The models with random effect for sites nested within a health authority were also considered; however, such effects did not improve the model fit according to the Bayes information criterion. Imputation of missing values on the CSQ-8 (*n* = 1) was performed by generating the empirical distributions for each variable and drawing a random number from that distribution to replace the missing value. Cases with half or more missing items on the CSQ-8 were not imputed, resulting in four cases being excluded, reducing the final sample to 119. Due to missing data on covariates, different numbers of participants were included in different models: *n* = 108 in Model 1, *n* = 108 in Model 2 and *n* = 74 in Model 3. To avoid the potential bias from the exclusion of participants with missing values, regression models with normalized inverse probability weights were estimated.

Multicollinearity was assessed using correlation analysis, and no issues were detected. Other assumptions of the linear mixed model were assessed through a standard residual diagnostic, and no major deviations were identified.

All tests were two-sided, and results with p-values < 0.05 were considered statistically significant.

## Results

The clinical and socio-demographic characteristics of the participants are shown in Table 1.

**Table 1:**
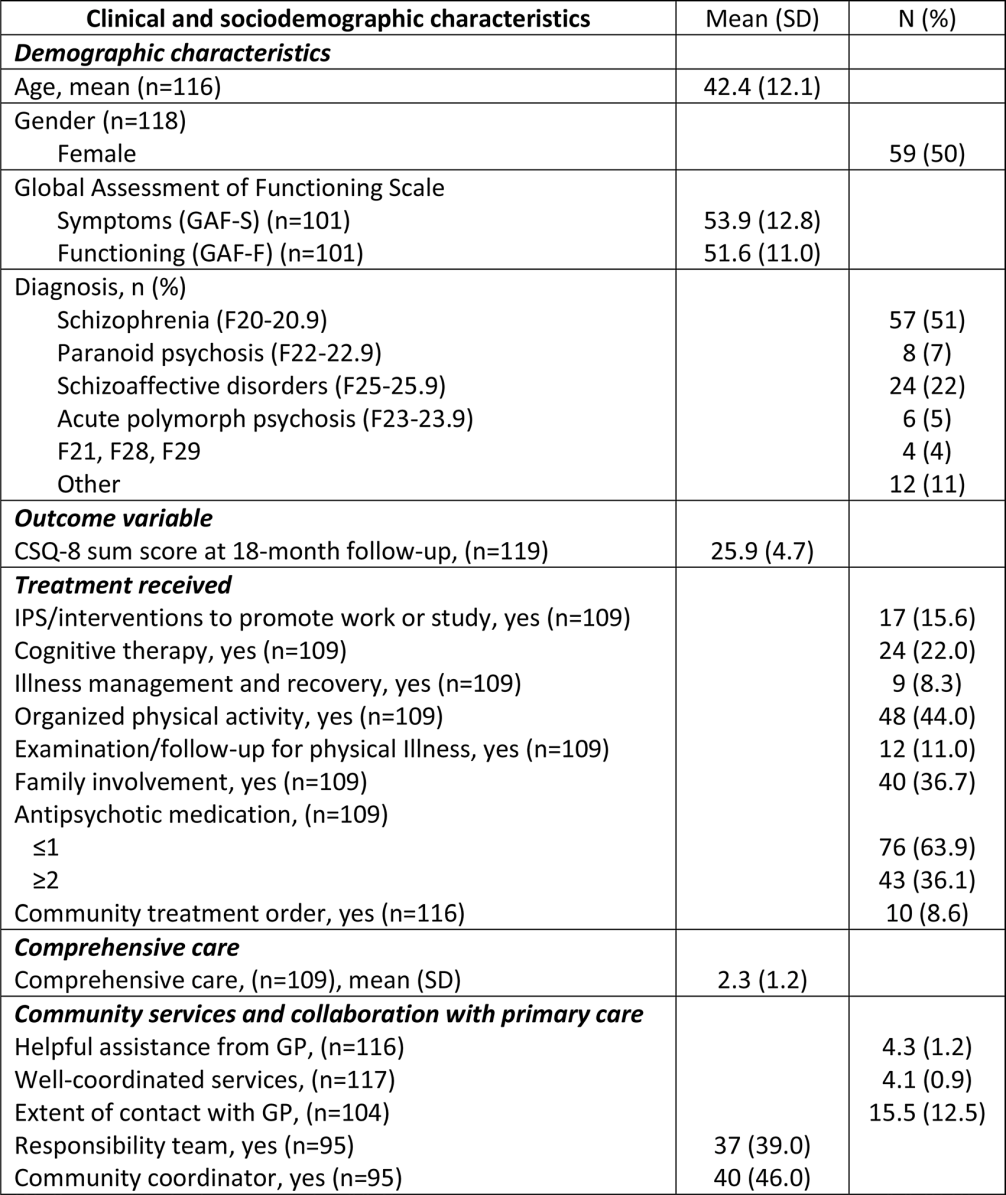
Clinical and sociodemographic characteristics of participants (*n* = 119)

### Dropout Analysis

Compared with the valid sample (*N* = 119), the drop-out sample (*n* = 206) included a significantly larger proportion of men (*p* = 0.013), a larger proportion having received cognitive therapy (*p* = 0.029), a higher proportion having undergone examination or follow-up for physical illness (*p* < 0.001), lower rates of having received helpful assistance from their GP (*p* < 0.001), and lower rates of having well-coordinated services (*p* = 0.028).

### Association Between Treatments Received at Baseline and Service Users’ Satisfaction with Mental Health Services at 18-Month Follow-up

Table 2 presents the results of the linear mixed model assessing the associations between the covariates representing treatments received at baseline and satisfaction with services (CSQ-8) at 18-month follow-up (Model 1). In both the adjusted and unadjusted models, participation in IPS/interventions to promote work or study was the only intervention associated with service satisfaction.

**Table 2:**
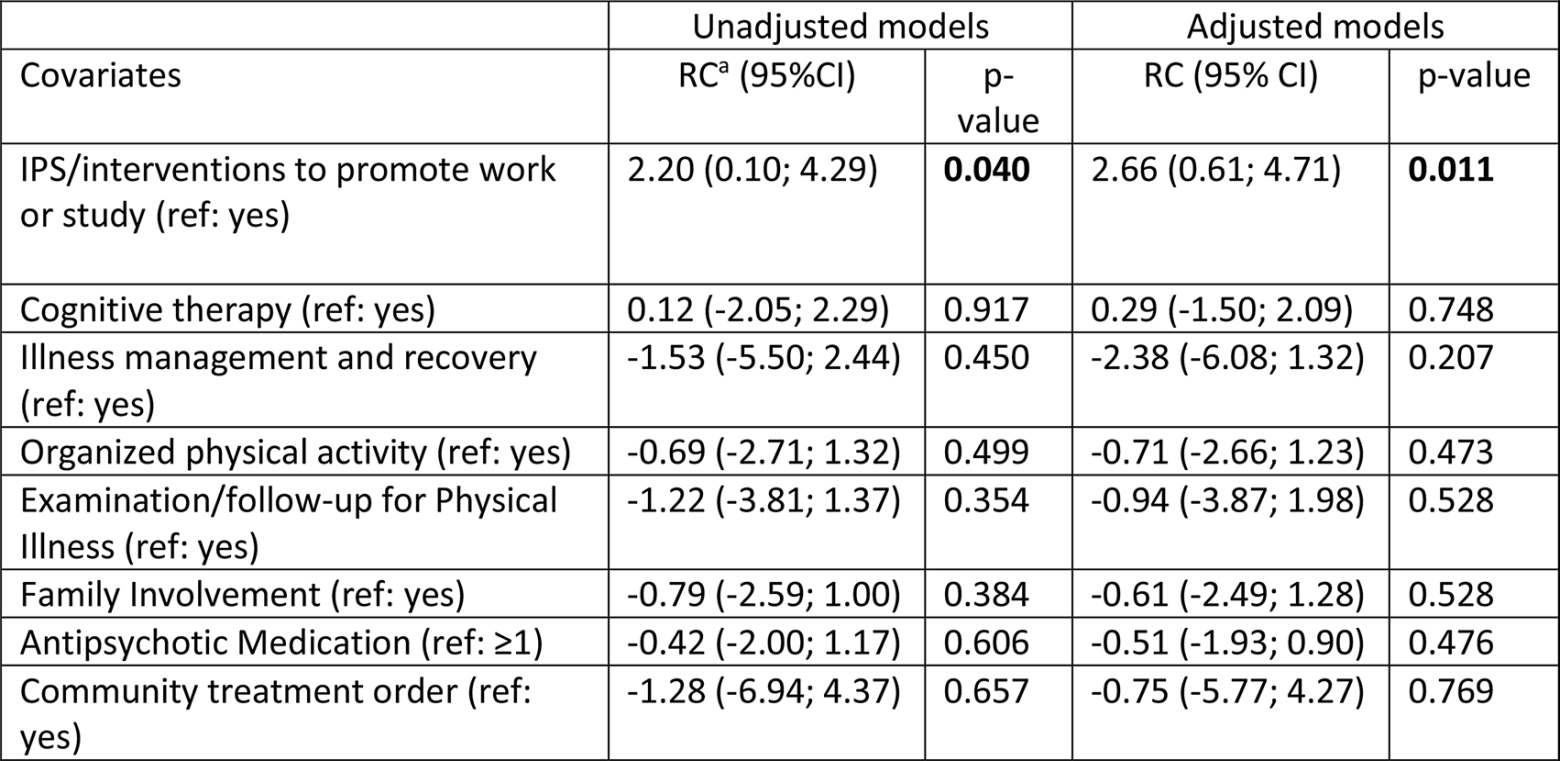
Linear mixed model results for associations between treatments received for the last 6 months as reported at baseline and satisfaction with services (CSQ-8) at 18-month follow-up. (model 1, *n* = 108)

^a^Regression Coefficient

### Associations Between Comprehensive Care at Baseline and Service Users’ Satisfaction with Mental Health Services at 18-Month Follow-up

Table 3 shows the results of the linear mixed model analysis assessing the associations between the comprehensive care variable at baseline and satisfaction with services (CSQ-8) at 18 months follow-up.

**Table 3:**
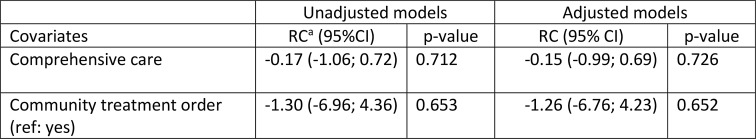
Linear mixed model results for associations between comprehensive care at baseline and satisfaction with services (CSQ-8) at 18-month follow-up and (model 2, *n* = 108)

^a^Regression Coefficient

### Associations Between Community Services and Collaboration with Primary Care at Baseline and Service Users’ Satisfaction with Mental Health Services at 18-Month Follow-up

Table 4 presents the results of the linear mixed model assessing the associations between covariates comprising community services and collaboration with primary care and satisfaction with services (CSQ-8). In both the adjusted and unadjusted models, service users’ experiences of receiving well-coordinated services and perceptions of receiving helpful assistance from a GP were associated with higher service satisfaction.

**Table 4:**
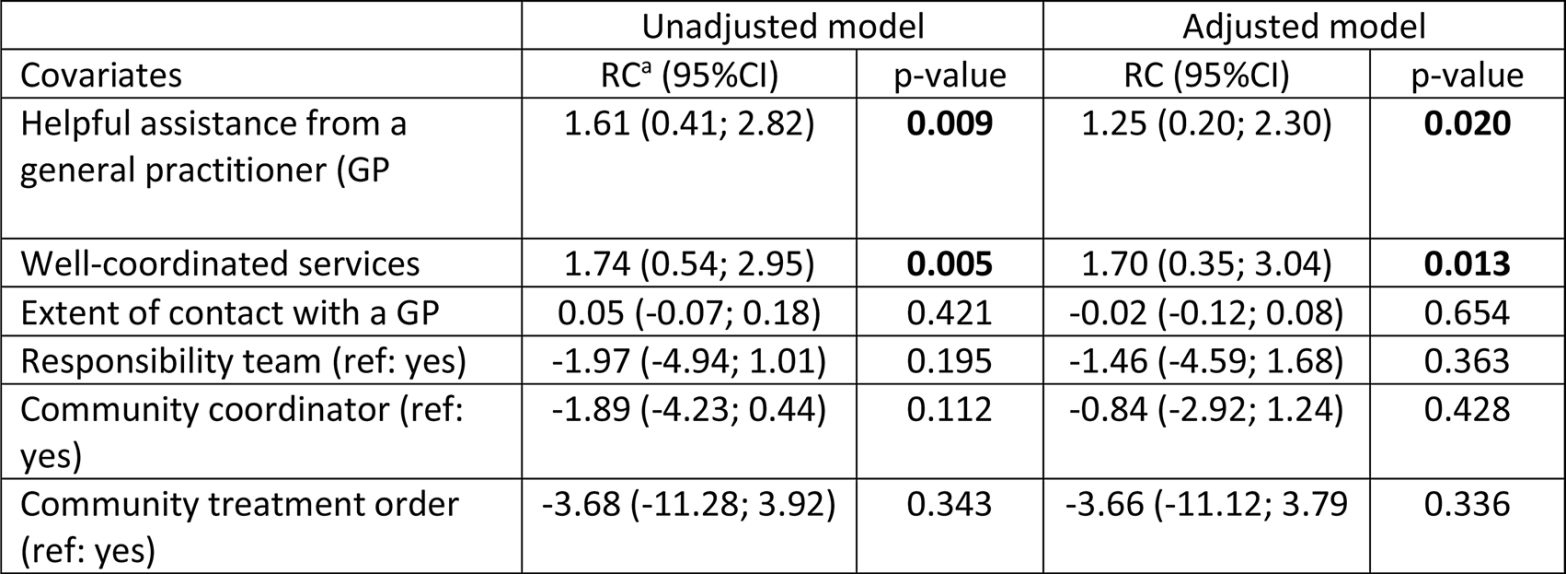
Linear mixed model results for associations between satisfaction with services (CSQ-8) and covariates on community services and collaboration with primary care (model 3, *n* = 74)

^a^Regression Coefficient

## Discussion

The results of this study indicated that service users’ satisfaction with services was positively associated with participation in the IPS program or other interventions to promote work or study, as well as with service users’ experiences of receiving well-coordinated services and perception of receiving helpful assistance from a GP. The well-known confounder in service satisfaction studies, being under involuntary care, was not associated with service satisfaction in our models.

IPS is an evidence-based practice for supporting people experiencing mental illness to obtain and maintain competitive employment (Bond et al., 2012). It builds on the acknowledgement that people with mental illness have the right to participate fully in community life, including regular employment (Bond et al., 2020). Participation in IPS programs has been shown to be associated with high satisfaction with the IPS program (Viering et al., 2015) and occupational situations (Nygren et al., 2011). One possible explanation for why IPS is associated with higher satisfaction may be that it provides individuals with specific goals to work toward, plays a role in normalizing and destigmatizing mental illness through promoting social inclusion, and may lead to an experience of agency. Being involved in work or study may also have an impact on the self-stigma often associated with mental illness. While many approaches in mental health services are primarily conducted in a therapy room, IPS involves active participation in real-world activities and settings, which can cultivate a sense of mastery and achievement (Bond et al., 2020).

The finding that service users’ experiences of receiving well-coordinated services and helpful assistance from their GP were associated with higher satisfaction is consistent with previous studies, which highlights the significance of receiving assistance or care outside mental health services (Ruud et al., 2016). Thus, the present results emphasize the importance of interdisciplinary collaboration. When services are well-coordinated, service users may feel less isolated in that they do not have to face their challenges alone. Effective communication among services reduces confusion for service users (Flatau et al., 2013) and ensures that everyone receives the same information (Rollins et al., 2017). Additionally, our results underscore the importance of having a dedicated GP, and this is in-line with previous research demonstrating the importance of a strong therapeutic relationship with GPs among individuals with severe mental illness (Light et al., 2015). GPs often serve as the primary point of contact outside mental health services, play a significant role in the lives of people with severe mental illness and often facilitate the management of service users’ mental health (Light et al., 2015). A previous Norwegian study reported a dose-response association between the duration of the relationship between the patient and the GP and clinical outcomes such as fewer acute hospital admissions (Sandvik et al., 2022). However, in the present, no associations were found between the extent of contact with the GP and satisfaction with services.

Our results underscore the importance of providing integrated and well-coordinated services to promote satisfaction among service users with the services and assistance they receive. This involves ensuring that each service user has a dedicated GP. Additionally, the IPS intervention is another valuable approach that extends help by specifically targeting the goal of securing regular employment, which holds significant meaning in an individual’s everyday life.

## Strength and Limitations

A major strength of this study is the use of the CSQ-8 as an outcome measure. The CSQ-8 has sound psychometric properties and has been used in a range of settings and populations. Another strength is the broad group of participants with psychosis who were recruited from ‘real world’ clinical practice in many different specialized units, which increases the generalizability of the results. However, as the study participants were not randomly selected, the sample might not be representative of the general patient population with psychosis. Although the clinicians responsible for participant recruitment were instructed to approach all eligible service users, we lack information on the actual number of individuals who were invited to participate in the study. Therefore, the extent to which our findings can be generalized to further patient populations may be limited. Further, many participants who agreed to participate at baseline were lost to follow-up, potentially leading to attrition bias. The dropout analysis showed that the dropout sample included a larger proportion of men, those who had undergone examination or follow-up for physical illness and those who had received cognitive therapy. The analysis also showed that compared with the final valid sample, the drop-out sample had significant lower rates of having received helpful assistance from their GP and having well-coordinated services. These finding suggest that the associations found in the valid sample could have been both weaker and stronger if those who dropped out had been included in the valid sample. This also weakens the generalizability of results.

Finally, we do not know to what extent the service users participated in the different interventions/treatments. Therefore, whether the extent of the various interventions might have impacted the level of satisfaction remains unknown. Additionally, the difference in the numbers of participants in each intervention, such as the low number of participants in the IMR group, could increase the risk of a type II error.

## Conclusions

In the present study, service users with psychosis who reported that the services they received were well-coordinated, that they received helpful assistance from their GP and who participated in an IPS or other interventions to promote work or study were significantly more satisfied with services after 18 months than the other participants. These findings underscore the importance of providing integrated and well-coordinated services and targeting the goal of facilitating employment and study opportunities among service users with psychosis. Regarding the implications of these findings, further efforts to develop and implement programs to facilitate work interventions should be made, and the collaboration and coordination of services should be even more emphasized than at present.

## Data Availability

The datasets used and/or analyzed during the current study are available from the corresponding author on reasonable request.
